# Triagem Familiar no Diagnóstico da Síndrome do QT Curto após Morte Súbita Cardíaca como Primeira Manifestação em Jovens Irmãos

**DOI:** 10.36660/abc.20200274

**Published:** 2021-07-14

**Authors:** Guilherme Augusto Teodoro Athayde, Natália Quintella Sangiorgi Olivetti, Francisco Carlos da Costa Darrieux, Luciana Sacilotto, Gabrielle D’Arezzo Pessente, Maurício Ibrahim Scanavacca

**Affiliations:** 1 Hospital das Clínicas da Faculdade de Medicina da Universidade de São Paulo Instituto do Coração São PauloSP Brasil Instituto do Coração do Hospital das Clínicas da Faculdade de Medicina da Universidade de São Paulo, São Paulo, SP - Brasil.

**Keywords:** Síndrome do QT Curto, Morte Súbita Cardíaca, Cardiodesfibrilador Implantável, Teste Genético, Canalopatias Cardíacas

## Introdução

Em 2000, Gussak et al.,^1^ publicaram uma série de casos em que taquiarritmias atriais, síncope e morte súbita cardíaca (MSC) se associavam a um intervalo QT pronunciadamente curto, sendo os primeiros a correlacionar todos esses achados em uma síndrome. Desde então, esforços vêm sendo realizados, a fim de melhor entender o comportamento e encontrar alternativas de tratamento para a síndrome do QT curto (SQTC); no entanto, esbarram na raridade da doença, na dificuldade em estabelecer parâmetros diagnósticos e na sua complexa manifestação clínica. Este artigo relata o caso de irmãos jovens com MSC e SQTC e versa sobre as dificuldades diagnósticas e terapêuticas da síndrome.

## Relato de Caso

Um indivíduo de 18 anos de idade apresentou morte súbita enquanto tinha relações sexuais. A necrópsia realizada não evidenciou uma causa *mortis* precisa e não havia alterações cardíacas estruturais. Após 6 meses do ocorrido, seu irmão, aos 11 anos de idade, enquanto caminhava voltando da escola, apresentou MSC revertida, sem evidência do ritmo, sendo levado à unidade de terapia intensiva (UTI) de um hospital da região, falecendo após 9 dias. A necrópsia, mais uma vez, demonstrou um coração estruturalmente normal.

**Figura 1:**
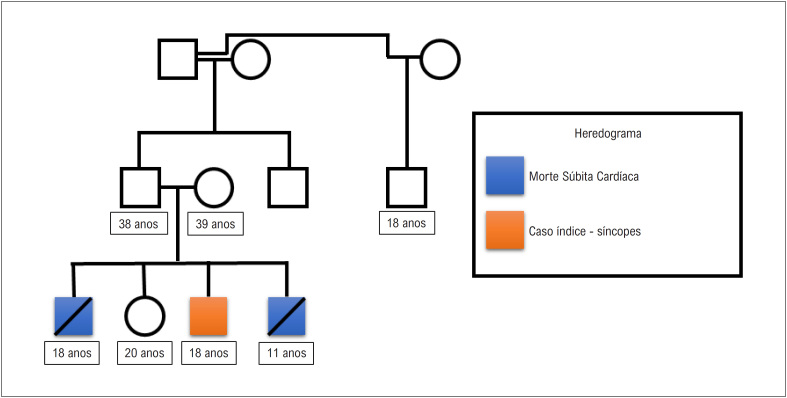
Heredograma familiar, mostrando o caso-índice em laranja com manifestação de síncopes. Seus irmãos, marcados em azul, sofreram morte súbita cardíaca aos 18 anos de idade (durante atividade sexual) e aos 11 anos de idade (caminhando de volta da escola), respectivamente, da esquerda para a direita. Morte Súbita cardíaca definida conforme Priori et al:^4^ nenhuma causa extracardíaca óbvia ocorreu no exame post mortem e, portanto, um evento arrítmico é a causa provável da morte.

**Figura 2:**
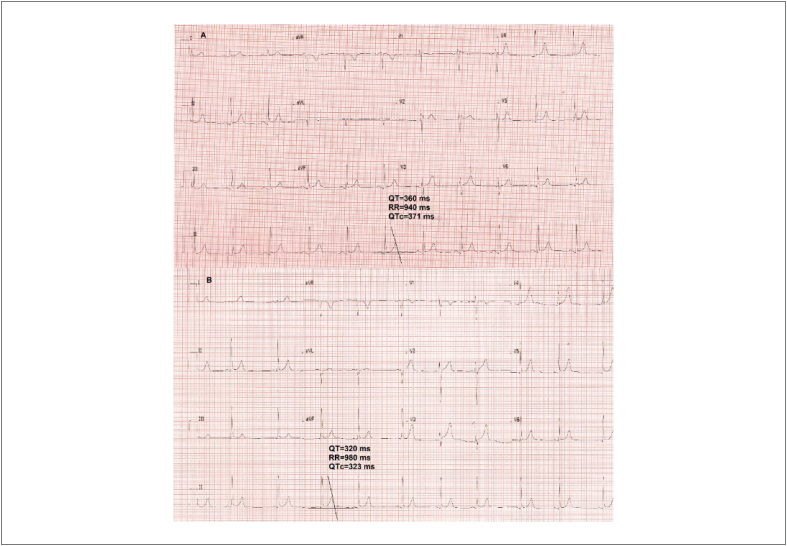
Eletrocardiogramas da chegada dos pacientes ao serviço, mostrando intervalos QT curtos. A. Eletrocardiograma do pai, com QTc = 371 ms. B. Eletrocardiograma do filho vivo, com QTc = 323 ms.

Seus familiares (Figura 1) procuraram auxílio médico para investigação diagnóstica. A história clínica revelava um passado de drogadição longínquo do pai, e o filho vivo de 18 anos apresentara síncopes sem pródromos na infância, sem relação com fatores desencadeantes sugestivos de fenômenos autonômicos. A filha viva, de 20 anos de idade, relatava palpitações com segundos de duração e tinha hipotireoidismo, assim como a mãe. O pai, o filho e o avô paterno apresentavam *pectus excavatum* e uma envergadura maior que a altura, todavia sem critérios para diagnóstico de síndrome de Marfan. O eletrocardiograma (ECG) do pai mostrava um QTc de 371 ms e o do filho vivo, de 323 ms (Figura 2), enquanto o da filha viva, QTc de 380 ms. Os exames ecocardiograma, ECG de alta resolução (ECG-AR), angiotomografia de aorta e ressonância magnética cardíaca de todos os familiares de primeiro grau encontravam-se sem alterações. Não se observaram arritmias atriais ou ventriculares no teste ergométrico ou no Holter de 24 horas.

Foi realizado teste genético do pai por sequenciamento de nova geração (NGS), não sendo identificadas variantes patogênicas ou possivelmente patogênicas em um painel de 101 genes que se correlacionam com MSC, incluindo SQTC (KCNH2, KCNQ1, KCNJ2, CACNA1C e CACNB2) e Marfan (FBN1 e TGFBR).

Na ausência de monitor de eventos implantáveis no sistema único de saúde, optou-se por implante de cardiodesfibrilador implantável (CDI) no filho de 18 anos, devido à história de síncopes sem pródromos, associada ao histórico familiar de MSC, e ao intervalo QTc encurtado, favoráveis ao diagnóstico de SQTC. Em seguimento de 18 meses, não apresentou síncope ou terapias pelo CDI.

## Discussão

A MSC em jovens com coração estruturalmente normal definido por necrópsia pode ser considerada presumivelmente arrítmica, especificamente por canalopatias cardíacas.^2^ As principais canalopatias incluem síndrome do QT longo, síndrome de Brugada, taquicardia ventricular polimórfica catecolaminérgica, doença progressiva da condução cardíaca e, por fim, SQTC.

A maioria desses distúrbios está associada a mutações em genes que codificam as subunidades alfaformadoras de poros do canal e suas interações com proteínas auxiliares, responsáveis pelo funcionamento dos canais de sódio, potássio e cálcio.^2^ Portanto, a avaliação de familiares em um contexto de duas MSC em jovens com necrópsia normal abrange análise clínica extensa dos sobreviventes, bem como testes genéticos *post mortem*.

O único achado relevante na investigação cínica familiar, especificamente no filho sobrevivente que apresentava síncope “desliga-liga” e no pai, foi um intervalo QT encurtado. O diagnóstico da SQTC é alvo de debate na literatura até os dias atuais. Os critérios propostos por Gollob et al.,^3^ em 2011, foram os primeiros a ser utilizados.^3^ Em 2015, novos critérios foram propostos,^4^ sendo a SQTC diagnosticada quando o intervalo QTc ≤340 ms ou quando o intervalo QTc <360 ms, em associação à história clínica, familiar ou presença de mutação patogênica. No caso exposto, o ECG do filho sobrevivente apresentava um QTc de 323 ms, associado à forte história de MSC em familiares ≤40 anos de idade, além do passado de síncopes inexplicadas. Isoladamente, um intervalo QTc <340ms é diagnóstico de SQTC e, pelo escore de Gollob, somaria 5 pontos,^3^ preenchendo critérios de alta probabilidade diagnóstica para a SQTC. Não documentamos fibrilação atrial até o momento, que reforçaria o diagnóstico de SQTC.

A morte súbita em irmãos jovens, sem antecedentes familiares adicionais de MSC, sugere a presença de doença recessiva, porém os pais não eram consanguíneos. Outra possibilidade é a ocorrência de mutação *de novo* em um dos genitores, com uma expressão clínica mais branda. O pai apresentava intervalo QT mais curto que a média populacional, porém era assintomático. Como uma doença autossômica dominante de penetrância incompleta e expressividade variável,^5^ ele poderia ser um carreador assintomático de uma canalopatia cardíaca expressa com maior gravidade nos filhos que faleceram. Além disso, as mortes e a síncope do sobrevivente aconteceram na segunda década de vida, mais sugestivos de TVPC, não identificada na avaliação clínica e genética.^4^

A real prevalência da SQTC é duvidosa em virtude de sua raridade. O encurtamento do intervalo QT reflete a repolarização acelerada, gerando uma dispersão da repolarização entre as camadas cardíacas, favorecendo o mecanismo de reentrada funcional nos átrios e ventrículos, predispondo à fibrilação atrial e ventricular.^6^

Em canalopatias, o teste genético tem papel limitado na síndrome de Brugada e na SQTC (cerca de 25%),^3^^,^^7^ diferentemente da SQTL (80%)^8^ e da TVPC (90%).^9^ Logo, um teste genético sem identificação de mutações patogênicas ocorre com mais frequência na SQTC^3^ e não exclui o diagnóstico de SQTC.

O CDI tem se mostrado como o tratamento mais efetivo e seguro para os pacientes com SQTC, uma vez que estes apresentam alto risco de MSC.^10^ O implante de CDI é recomendado para pacientes com diagnóstico de SQTC que são sobreviventes de uma MSC ou que têm taquicardia ventricular (TV) sustentada espontânea documentada.^4^ No nosso caso, o tratamento proposto foi o implante de CDI no filho vivo e a observação clínica do restante da família, de acordo com as recomendações mais recentes que sugerem que um CDI poderia ser considerado em pacientes com SQTC com uma forte história familiar de MSC e uma evidência de QTc curto, como classe de recomendação IIb.^4^

## Conclusão

O diagnóstico etiológico da MSC em jovens pode ser desafiador, principalmente quando há suspeita de morte súbita presumivelmente arrítmica, por sua característica de penetrância incompleta e expressividade variável. A síndrome do QT curto é uma canalopatia muito rara e, portanto, com uma caracterização fenotípica limitada, que deve ser recordada no cenário de MSC de etiologia desconhecida em jovens. Seu diagnóstico baseia-se apenas no ECG, na história clínica e familiar e na genética. O CDI é a principal arma terapêutica, mostrando-se um tratamento efetivo e seguro para a redução da mortalidade nesses pacientes.
