# Supplementary material for: Triagem Familiar no Diagnóstico da Síndrome do QT Curto após Morte Súbita Cardíaca como Primeira Manifestação em Jovens Irmãos
Source: Arq Bras Cardiol. 2021 Jul 14;117(1 Supl 1):12–5. [Article in Portuguese] doi: 10.36660/abc.20200274 (PMC8291896; doi:10.36660/abc.20200274)
Supplement: Translation

# Translation: Family Screening in the Diagnosis of Short QT Syndrome after Sudden Cardiac Death as First Manifestation in Young Siblings

**Authors:** Guilherme Augusto Teodoro Athayde, Natália Quintella Sangiorgi Olivetti, Francisco Carlos da Costa Darrieux, Luciana Sacilotto, Gabrielle D’Arezzo Pessente, Maurício Ibrahim Scanavacca

**Affiliations:** 1 São PauloSP Brazil Instituto do Coração do Hospital das Clínicas da Faculdade de Medicina da Universidade de São Paulo, São Paulo, SP - Brazil.

**Keywords:** Short QT Syndrome, Sudden Cardiac Death, Implantable Cardioverter-defibrillator, Genetic Testing, Cardiac Channelopathies

## Introduction

In 2000, Gussak et al.,^1^ published a case series in which atrial tachyarrhythmias, syncope, and sudden cardiac death (SCD) were associated with a short QT interval, being the first to correlate all of these finding in one single study. Since then, efforts have been made to better understand the behavior of and find alternatives to treat short QT syndrome (SQTS); however, researchers face the barrier of the rarity of the disease, the difficulty in establishing diagnostic parameters, and its complex clinical manifestation. This article reports on the case of young siblings with SCD and SQTS and discusses the syndrome's diagnostic and therapeutic difficulties.

## Case Report

An 18 year old boy had a sudden death during sexual intercourse. Autopsy examination was within normal. After six months, his young brother, at 11 years old, had an aborted cardiac arrest while walking home from school. He died after 9 days in a local hospital. The autopsy, once again, was inconclusive.

Their family members (Figure 1) sought out medical assistance to conduct a diagnostic investigation. Their medical history revealed the father's long-term drug addiction and the 18-year-old son. had a past history, during childhood, of syncope without prodromes, no triggered by autonomic circunstances. The 20-year-old daughter had hypothyroidism and reported palpitations, lasting seconds. The mother also reported the medical condition of hypothyroidism and her cardiac evaluation was normal. The father, the son, and the grandfather presented *pectus excavatum* and an increased arm span, without fulfilling the criteria for Marfan Syndrome. The electrocardiogram (ECG) of the father showed a QTc of 371 ms and of the living son of 323 ms (Figure 2), while the living daughter presented a QTc of 380 ms. The high resolution ECG (HR-ECG), aorta angiotomography, and of all of the first degree relatives were within normal. No atrial or ventricular arrhythmias were observed in the stresse testing or in the 24-hour Holter monitoring.

**Figure 1:** Family Heredogram, showing the index case in orange with the manifestation of syncopes. Their brothers, marked in blue, suffered sudden cardiac death at 18 years old (during sexual intercourse) and at 11 years old (walking home from school), respectively, from left to right. Sudden cardiac death defined according to Priori et al:^4^ no obvious extra-cardiac cause occurred in the post-mortem exam and, therefore, an arrhythmic event is the most probable cause of death.

**Figure 2:** Electrocardiogram of the arrival of the patients to the health service, showing short QT intervals. A. Electrocardiogram of the father, with QTc = 371 ms. B. Electrocardiogram of the living son, with QTc = 323 ms.

A Next-Generation Sequencing (NGS) test of the father was performed, and no pathogenic or possibly pathogenic variants were identified in a panel of 101 genes correlated with SCD, including SQTS (KCNH2, KCNQ1, KCNJ2, CACNA1C, and CACNB2) and Marfan (FBN1 and TGFBR).

Due to the implanted loop recording unavailability in the Brazilian Unified Health System (SUS, in Portuguese), we opted for the implantable cardioverter defibrillator (ICD) in the 18-year-old son, due to his medical history of syncopes without prodromes, associated with a family history of SCD and short QTc interval, favorable to the diagnosis of SQTS. In an 18-month follow-up, the patient presented no syncope or ICD therapy.

## Discussion

SCD in young patients with structurally normal hearts, defined by autopsy examination, can presumably be considered arrhythmic, specifically due to cardiac channelopathies.^2^ The main channelopathies include long QT syndrome, Brugada syndrome, catecholaminergic polymorphic ventricular tachycardia, progressive heart disease, and finally, SQTS.

Most of these disorders are associated with the gene mutations that codify the alphaform subunits of the channel pores and their interactions with auxiliary proteins, responsible for the functioning of the sodium, potassium, and calcium channels.^2^ Therefore, the evaluation of family members in a context of two SCDs in young individuals with inconclusive autopsy includes an extensive clinical analysis of the survivors, along with post-mortem genetic tests.

The only relevant finding in the familiar screening, specifically in the surviving son who presented a “off-on” syncope, as well as in the father, was a short QT interval. The SQTS diagnosis is the target of debate in the literature even today. The criteria proposed by Gollob et al.,^3^ in 2011, were the first to be implemented.^3^ In 2015, new criteria were proposed,^4^ with the SQTS diagnosed when the QTc interval ≤340 ms or when the QTc interval <360 ms, in association with the medical or family history or with the presence of pathogenic mutation. In the case explained above, the ECG of the surviving son presented a QTc of 323 ms, associated with a strong history of SCD in family members of ≤40 years of age, in addition to the past medical history of unexplained syncopes. In an isolated manner, a QTc interval <340ms is diagnosed as an SQTS and, by the Gollob score, would add up to 5 points,^3^ fulfilling high-probability diagnostic criteria for SQTS. To date, we have not documented atrial fibrillation that would reinforce the diagnosis of SQTS.

The sudden death in these two young brothers, without prior additional family medical history of SCD, suggests the presence of recessive disease, however parents were non-consanguineous. Paternity test was not performed, but the father short QTc interval and dismorphic features suggested a paternal inheritance. Another possibility is the occurrence of a de novo mutation in one of the parents, with a lighter clinical expression. The father presented a shorter QT interval than the general population average but was asymptomatic. As it is a dominant autosomal disease of incomplete penetrance and variable expressivity,^5^ the father might be a silent carrier. Moreover, the death and the syncope of the survivor occurred in the second decade of life, which is more common for other channelopathies.^4^ The clinical and genetic evaluation did not identify it.

The real prevalence of SQTS is doubtful due to its rarity. The short QT interval reflects accelerated repolarization, generating a dispersion of the repolarization within the heart chambers, favoring the mechanism of functional re-entrance in the atrium and ventricles, predisposing the patient to atrial and ventricular fibrillation.^6^

In channelopathies, the genetic test plays a limited role in the Brugada syndrome and in the SQTS (approximately 25%),^3^^,^^7^ differently from SQTL (80%)^8^ and from cathecolaminergic polymorphic ventricular tachycardia (CPVT) (90%).^9^ Hence, a genetic test without the identification of pathogenic mutations in patients with SQTS does not preclude the clinical diagnosis,^3^ and does not exclude the diagnosis of SQTS.

ICD has proven to be the most effective and safe treatment for SQTS patients, since it presents a high risk for SCD.^10^ The ICD is recommended for patients with SQTS diagnosis who are survivors of an SCD or who have a documented spontaneous sustained ventricular tachycardia.^4^ In our case, the treatment proposed was the ICD implant in the symptomatic son (with syncopal episodes) and close follow-up for the others, according to more recent recommendations, which suggest that the ICD should be considered in SQTS patients with a strong family history of SCD and evidence of short QTc as a IIb class of recommendation.^4^

## Conclusion

The etiological diagnosis of SCD in young patients can be challenging, especially when there is the suspicion of sudden arrhythmic death, due to its incomplete penetrance and variable expressivity. The QT syndrome is a very rare channelopathy and, therefore, with a limited phenotypical characterization, which should be taken into consideration in a scenario of SCD of unknown etiology in young patients. Its diagnosis is based only on the ECG, the medical and family history, and genetics. The ICD is the main therapeutic tool, proving to be an effective and safe treatment for the reduction of mortality in these patients.
